# Transcriptional reprogramming strategies and miRNA-mediated regulation networks of *Taxus media* induced into callus cells from tissues

**DOI:** 10.1186/s12864-020-6576-2

**Published:** 2020-02-18

**Authors:** Ying Chen, Meng Zhang, Xiaofei Jin, Haoran Tao, Yamin Wang, Bo Peng, Chunhua Fu, Longjiang Yu

**Affiliations:** 1 Institute of Resource Biology and Biotechnology, Department of Biotechnology, College of Life Science and Technology, Huazhong University of Science and Technology, No.1037 Luoyu Road, Wuhan, 430074 People’s Republic of China; 2 Key Laboratory of Molecular Biophysics Ministry of Education, College of Life Science and Technology, Huazhong University of Science and Technology, No.1037 Luoyu Road, Wuhan, 430074 People’s Republic of China; 3 Hubei Engineering Research Center for Edible and Medicinal Resources, Wuhan, 430074 People’s Republic of China

**Keywords:** *Taxus* callus, miRNA, Transcription factors, Taxol biosynthesis, Plant defenses

## Abstract

**Background:**

*Taxus* cells are a potential sustainable and environment-friendly source of taxol, but they have low survival ratios and slow grow rates. Despite these limitations, *Taxus* callus cells induced through 6 months of culture contain more taxol than their parent tissues. In this work, we utilized 6-month-old *Taxus media* calli to investigate their regulatory mechanisms of taxol biosynthesis by applying multiomics technologies. Our results provide insights into the adaptation strategies of *T. media* by transcriptional reprogramming when induced into calli from parent tissues.

**Results:**

Seven out of 12 known taxol, most of flavonoid and phenylpropanoid biosynthesis genes were significantly upregulated in callus cells relative to that in the parent tissue, thus indicating that secondary metabolism is significantly strengthened. The expression of genes involved in pathways metabolizing biological materials, such as amino acids and sugars, also dramatically increased because all nutrients are supplied from the medium. The expression level of 94.1% genes involved in photosynthesis significantly decreased. These results reveal that callus cells undergo transcriptional reprogramming and transition into heterotrophs. Interestingly, common defense and immune activities, such as “plant–pathogen interaction” and salicylic acid- and jasmonic acid-signaling transduction, were repressed in calli. Thus, it’s an intelligent adaption strategy to use secondary metabolites as a cost-effective defense system. MiRNA- and degradome-sequencing results showed the involvement of a precise regulatory network in the miRNA-mediated transcriptional reprogramming of calli. MiRNAs act as direct regulators to enhance the metabolism of biological substances and repress defense activities. Given that only 17 genes of secondary metabolite biosynthesis were effectively regulated, miRNAs are likely to play intermediate roles in the biosynthesis of secondary metabolites by regulating transcriptional factors (TFs), such as ERF, WRKY, and SPL.

**Conclusion:**

Our results suggest that increasing the biosynthesis of taxol and other secondary metabolites is an active regulatory measure of calli to adapt to heterotrophic culture, and this alteration mainly involved direct and indirect miRNA-induced transcriptional reprogramming. These results expand our understanding of the relationships among the metabolism of biological substances, the biosynthesis of secondary metabolites, and defense systems. They also provide a series of candidate miRNAs and transcription factors for taxol biosynthesis.

## Background

Taxol (generic name: paclitaxel), was first identified in the bark of *Taxus brevifolia* (Pacific yew) and is commonly used as a clinical drug for several types of cancer, such as lung cancer, and other solid tumors [1]. The substance is mainly extracted from the leaves and stems of *Taxus* spp., such as *Taxus media*. *T. media* is the natural hybrid of the maternal *Taxus cuspidata* and paternal *Taxus baccata* and contains considerable amounts of taxol in nearly all of its tissues [2]. However, although *T. media* has been extensively cultivated, current taxol supplies cannot meet clinical requirements due to limited land resources and the low content of the desired products in the collected plant tissues.

Induction of callus cells of *Taxus* spp. is considered a promising means to produce taxol and can prevent the severe misuse of conventional plant species [3]. However, most *Taxus* calli newly induced from tissues turn brown and cease to grow within 6–12 months, and this tissue often requires approximately 1–2 years or more to grow for industrial use [4]. Cells cultured through long-term suspension subculture show drastically reduced taxol yields and exhibit numerous other problems, such as heterogeneity and cellular ploidy [5–7]. These issues inhibit the extensive industrial use of *Taxus* cells for taxol production. Therefore, clarifying the regulatory mechanism of taxol biosynthesis is beneficial to address these challenges.

In a previous study, we investigated the long-term (10 years) subculture of *Taxus* cells and found that long-term subculture changes the living behavior of *Taxus* cells, resulting in reductions in taxol and other secondary metabolites and strengthening of their primary metabolism. These results indicate a vague regulatory mode in which long-term subcultured cells undergo decrease taxol biosynthesis [7].

*Taxus* suspension cells require approximately 1–2 years or more of subculture before they can be used for industrial culture. During the induction and formation of suspension cells, newly induced *Taxus* calli, which develop within 6 months of initiation of induction, contain more taxol than their parent tissues [4]. To clarify the regulatory mechanism of taxol biosynthesis in *Taxus* cells, we applied multiomics technology to compare callus cells with parent tissues.

Multiomics technologies have been applied to clarify complicated regulatory networks; these technologies can reveal the details of regulatory mechanisms and validate the results of high-throughput sequencing analysis [8–10]. MiRNAs have previously been recognized to be key regulators of taxol biosynthesis and transcriptional reprogramming in *Taxus* cells [7]. Moreover, several miRNAs regulate TFs and epigenetic factors in tissue dedifferentiation in many plants [11–13]. For example, Wu et al. found that miRNAs direct DNA methylation at loci in which they are produced, as well as in *trans* to their target genes, and play roles in gene regulation [14]. Shen et al. confirmed that miRNAs participate in dedifferentiation by regulating key functional genes enriched in the pathways of plant hormone signal transduction [15]. Therefore, miRNAs may be crucial factors in the regulatory mechanisms of taxol biosynthesis in callus cells.

In this work, multiomics analyses were conducted to detect the reprogrammed transcriptional profiles of newly induced callus cells, reveal the key pathways and factors influencing taxol biosynthesis and transcriptional alterations, and verify and screen key miRNAs and targets.

## Results

### RNA-, miRNA-, and degradome-sequencing datasets

**Fig. 1:**
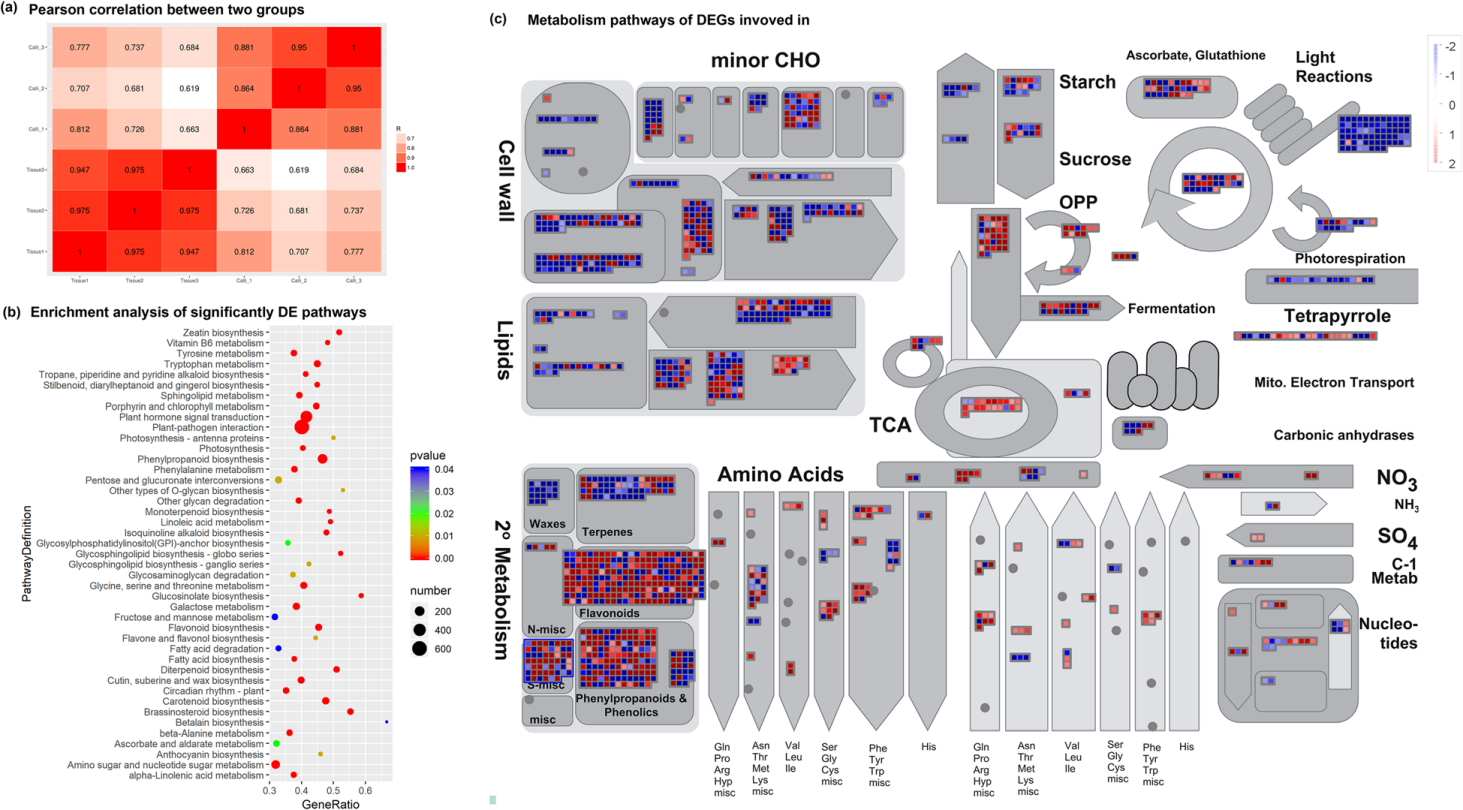
Transcriptional alterations in callus cells Total six samples from two groups were high-throughput sequenced. Six samples were validated by Pearson correlation analysis (**a**), and they were obviously separated into two groups, callus cells and tissues. All differentially expressed genes were annotated with KEGG database, and the significant DE pathways were showed in (**b**). These DEGs were analyzed by Mapman3.6.0, and a metabolism overview were showed in (**c**)

For RNA-seq, 66.69 Gb clean reads were obtained from two groups through three independent biorepeats; quality control assessment showed Q20 values of 96.66% (Table 1). Then, 74,603 unigenes with an N50 of 1464 bp were assembled, and the genes were annotated using the NR, Swiss-prot, COG, GO, and KEGG databases (Additional files 1 and 2, Table 2). Here, 11,956 unigenes were differentially expressed between the callus cells and tissues (Additional file 1d, Additional file 2, Table 2). Most differentially expressed genes (DEGs) were mainly located at the plasma membrane and the integral module of membranes (Additional file 3a, Additional file 4) and significantly involved in “plant–pathogen interaction” and “plant hormone signal transduction” (Fig. 1a, b, Additional file 5).

**Table 1:** Quality of clean reads

Sample	Raw_Reads	Raw_Bases (Gb)	Valid_Reads	Valid_Bases (Gb)	Valid%	Q20%	Q30%	GC%
Tissue1	8.1E+ 07	12.22	7.9E+ 07	11.44	97.46	96.99	92.39	44.93
Tissue2	6.8E+ 07	10.23	6.7E+ 07	9.79	98.25	98.02	94.76	44.83
Tissue3	8E+ 07	12.02	7.8E+ 07	11.33	97.76	97.30	93.03	45.13
Calli_1	7.4E+ 07	11.12	7.2E+ 07	10.36	97.41	96.76	91.80	46.46
Calli_2	8.6E+ 07	12.89	8.3E+ 07	11.93	96.93	96.66	91.62	45.73
Calli_3	8.4E+ 07	12.56	8.2E+ 07	11.84	97.74	97.33	93.01	46.27

**Table 2:** Quality of assemblies

Index	All	GC%	Min Length	Median Length	Max Length	Total Assembled Bases	N50
Transcript	127,215	41.15	201	503	17,640	114,510,759	1556
Gene	74,603	41.12	201	386	17,640	58,659,419	1464

Through miRNA-seq, 493 miRNAs were detected, 161 of which were newly identified in *T. media* (Additional file 6). A total of 95 miRNAs, including 35 novel miRNAs, were considered to be differentially expressed between the two groups with a *p*-value (Student T-test) of less than 0.05 (Fig. 1a, Additional files 7 and 8).

Degradome sequencing revealed that 1829 unigenes were degraded by 347 miRNAs, leading to 2432 degradation targets; of these, 323 unigenes were degraded by more than one miRNA (Additional file 9). Among the 347 identified miRNAs, cme-MIR166e-p5_2ss9CT19GC, mtr-MIR171c-p5_2ss1TC17GC, and ath-miR5021_R-1_1ss1TA degraded the most targets, specifically, 163, 93, and 84 unigenes, respectively. Among the degraded targets, two SPL-like TFs were degraded by the largest number of miRNAs, up to 11 (Additional file 9). SPLs (SQUAMOSA promoter-binding protein-like) form a plant-specific transcription factor family and participate in key activities; IPA1, for example, participates in the formation of plant architecture [16]. *SPLs* have recently been found to be tightly regulated by miRNAs in many plants, which indicates that they are crucial targets of miRNA and important nodes in the regulatory networks of plants [16–19].

**Fig. 2:**
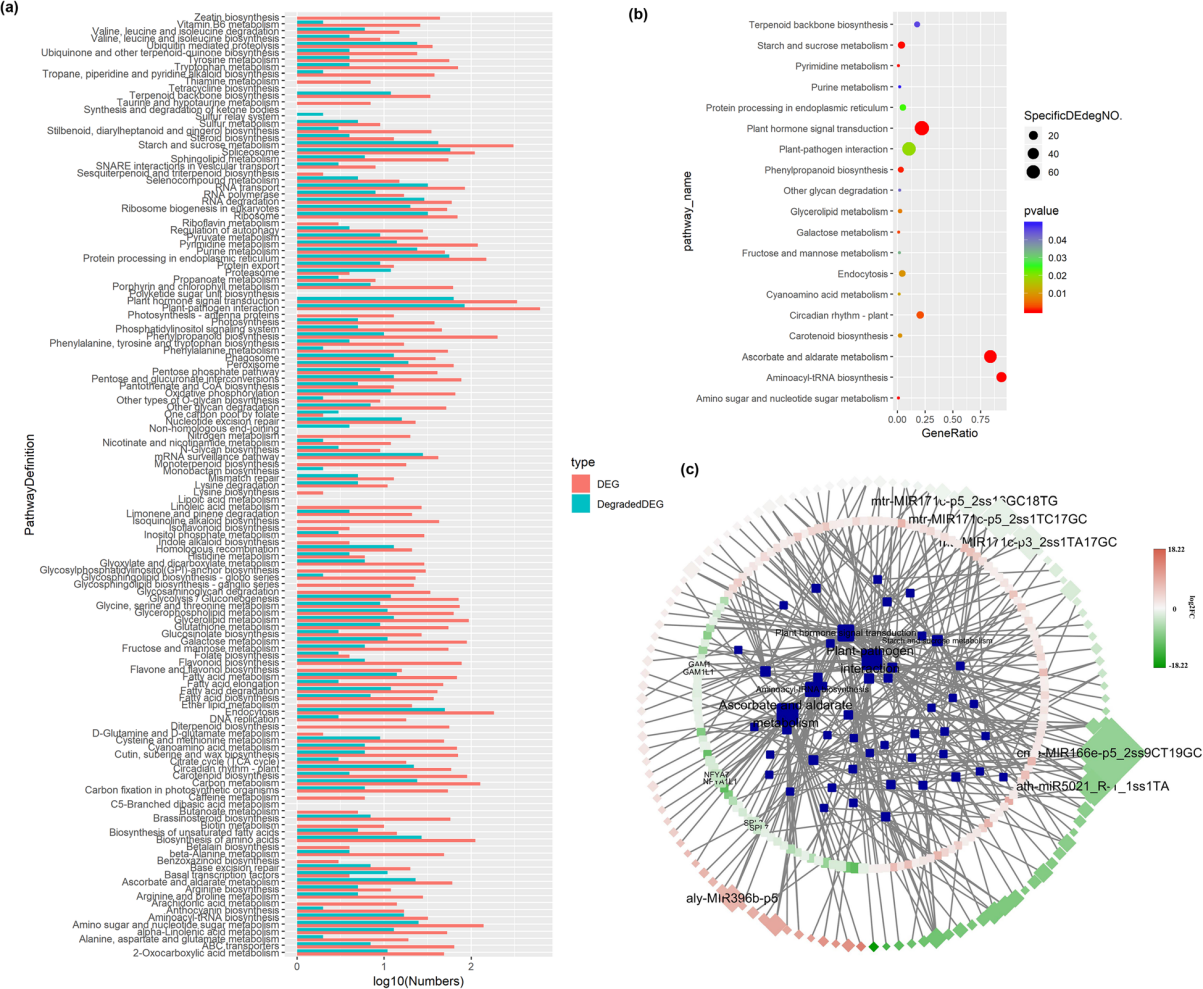
Function annotation of miRNA and degraded targets. Degraded DE genes were annotated to be involved in nearly all differentially expressed pathways (**a**). Moreover, the DE degraded genes significantly enriched in 19 pathways (**b**). Among the opposite expressed miRNAs and targets, there were six miRNAs and six degraded targets found to have the most targets and be targeted respectively (**c**). The DE miRNAs and their DE targets mostly enriched in 5 pathways, including “Plant-Pathogen Interaction”, “Plant Hormone signaling transduction”, “Ascorbate and aldarate metabolism”, “Starch and sucrose metabolism” and “Aminoacyl-tRNA biosynthesis”. FC was short for foldchange

Degraded differentially expressed (DE) targets were detected in approximately all DE pathways and significantly involved in 19 pathways; this result suggests that miRNAs are regulators involved in the transcriptional reprogramming of callus cells (Fig. 2a, Additional file 10).

### Callus cells are highly active in taxol biosynthesis but indirectly regulated by targeting miRNAs

**Fig. 3:**
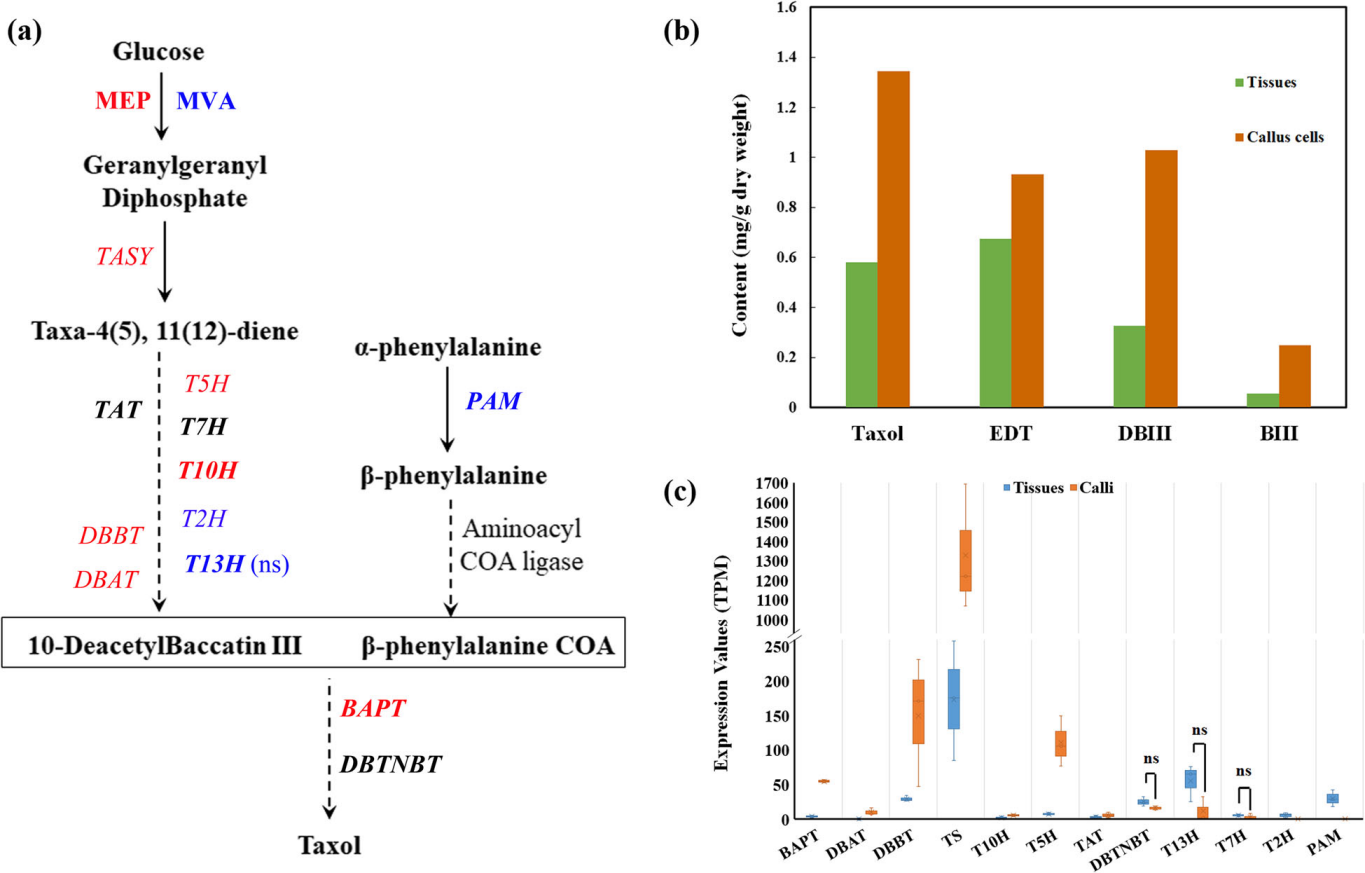
Taxol biosynthesis in callus cells and tissues Taxol biosynthesis was significantly upregulated in callus cells. **a** Taxol biosynthesis pathways. Genes/pathways in red indicated they were upregulated, blues were downregulated, darks had no differences. And bold gens mean they were targeted by miRNAs. MEP and MVA are short for Non-mevalonate pathway and Mevalonate pathway. Solid arrows mean the enzymatic step were certificated, while dotted arrows mean there were several unknown steps. **b** Taxanes content in callus cells and tissues. DBIII: 10-Deacetylbaccatin III, BIII: baccatin III, EDT: 10-deacetyl taxol. **c** Box-plot of expression values of taxol biosynthesis genes. Ns mean the different was not significantly in callus and tissues. TS (TASY): taxadiene synthase, DBAT: 10-deacetylbaccatin III-10-O-acetyl transferaseferase, PAM: phenylalanine ammonia-lyase, T5H: taxadiene 5-alpha hydroxylase, TAT: taxadienol acetyl transferase, T10H: 5-alpha-taxadienol-10-beta-hydroxylase, T13H: 13-alpha-hydroxylase gene, DBBT: taxane 2-alpha-O-benzoyltransferase, DBTNBT: 3′-N-debenzoyltaxol N-benzoyltransferase, BAPT: phenylpropanoyltransferase

The taxol content of newly induced callus cells was 1.34 mg/gDW (dry weight), which is 2.32 times higher than that in their parent tissues, and most biosynthesis genes were upregulated (Fig. 3). Additionally, the amount of 10-deacetylbaccatin III, an intermediate precursor of taxol, in callus cells was 1.02 mg/gDW; such content is 3.15-fold higher than the content of parent tissues. The contents of two other taxanes in callus cells, namely, 10-deacetyl taxol and baccatin III, were also high (Fig. 3b).

Taxol biosynthesis genes were found to be active, and 7 out of 12 known taxol biosynthesis genes were significantly upregulated in callus cells (Fig. 3a and c). The expression of the rate-limiting gene, *10-deacetylbaccatin III-10-O-acetyl transferase* (*DBAT*), which was barely expressed in tissues, increased by 70.9 times in callus cells. The expression of *Taxadiene synthase* (*TASY*), which is involved in the first step of taxol synthesis, increased by over 13.96-fold in callus cells; this gene also showed a high expression level in parent tissues. Five other genes, namely, *phenylpropanoyltransferase (BAPT)*, *taxadiene 5-alpha hydroxylase* (*T5H*), *taxane 2-alpha-O-benzoyltransferase* (*DBBT*), *5-alpha-taxadienol-10-beta-hydroxylase* (*T10H*), and *taxadienol acetyl transferase* (*TAT*), were significantly upregulated in callus cells (Fig. 3c). These results suggest that *TASY*, *DBAT*, *BAPT*, *T5H*, and *DBBT* are critically important for taxol biosynthesis.

Not all biosynthesis genes were upregulated in callus cells. *Phenylalanine ammonia-lyase* (*PAM*) and *taxane 2-alpha hydroxylase* (*T2H*) were downregulated; in particular, the former was barely detectable in callus cells (Fig. 3c). The functions of these two genes requires further elucidation.

**Fig. 4:**
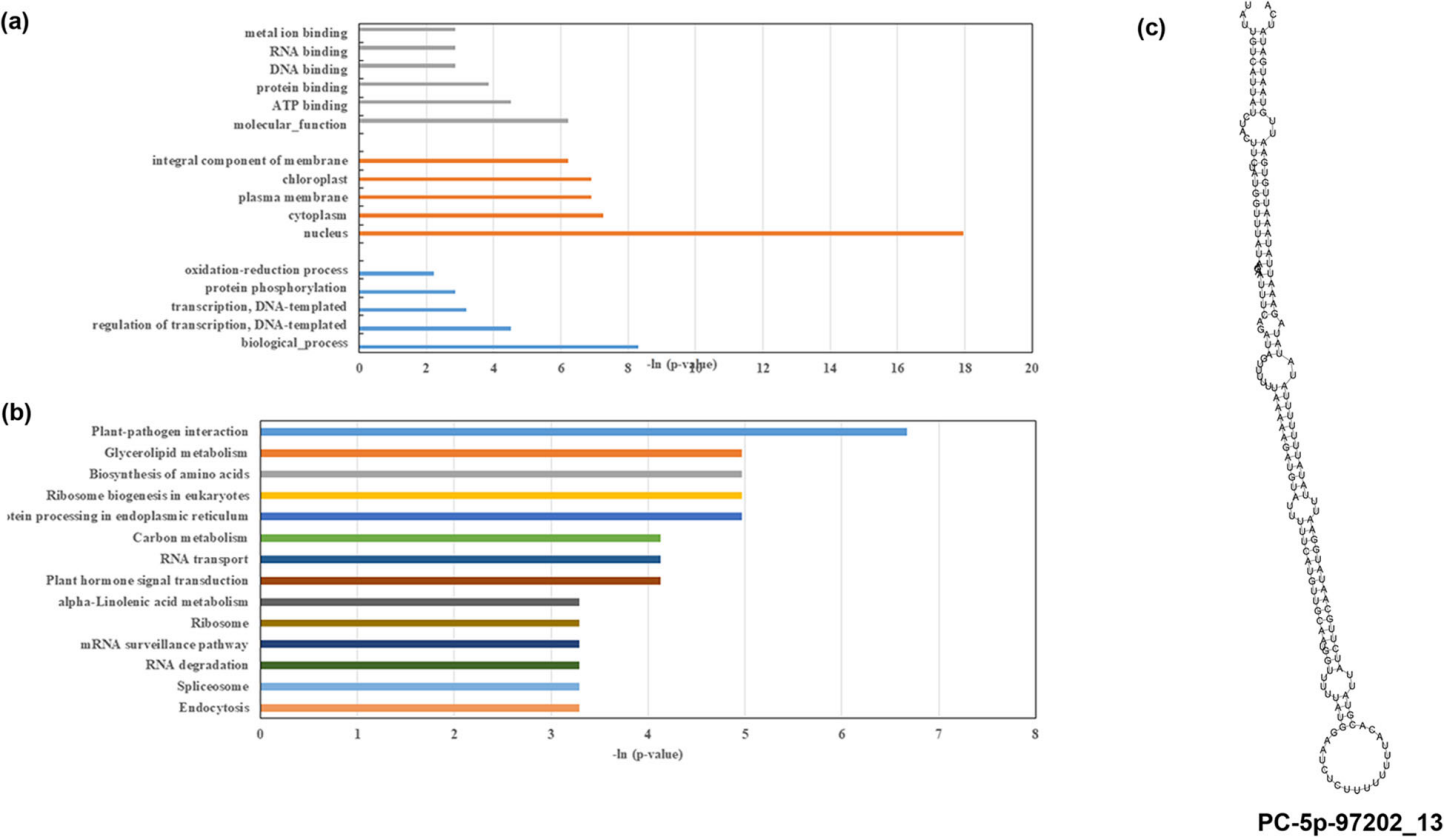
Annotation of degraded targets of 10 miRNAs. These 10 miRNAs, which targeted to taxol biosynthesis genes, degraded 226 genes totally. And these degraded targets were functional annotated with GO (**a**) and KEGG (**b**), they were significantly involved in the pathways that callus cells mainly transcriptional reprogrammed. The pre-miRNA of Pc-5p-97202_13, which targeted to taxol biosynthesis genes was predicted to form a stable hairpin in structure (**c**)

Seven homologue genes of *T7H*, *DBBT*, *BAPT*, *TAT*, *T10H*, *T13H*, *PAM*, and *DBTNBT* were targeted by 10 miRNAs, none of which were differentially expressed (Fig. 3a). *T7H*, *DBBT*, and *BAPT* are targeted by several miRNAs [7, 20–22]. Here, for the first time, *BAPT* was found to be targeted by miRNAs; *T10H* was targeted by miR5248 and miR397a, whereas *BAPT* was targeted by gma-miR6300 and the *Taxus*-specific miRNA PC-5p-97202_13 (Fig. 4c). Moreover, PC-5p-97202_13 was identified in *Taxus* spp. for the first time and found to degrade 34 targets (Fig. 4c, Additional file 11).

Degradome sequencing revealed that no taxol biosynthesis genes were degraded by any miRNA; however, a homologue of *T10H* was degraded by mtr-miR5248_2ss6AT21AT (Additional file 11). While *T10H-like* was significantly upregulated by 4.08-fold in callus cells, the expression of mtr-miR5248_2ss6AT21AT did not differ, thus suggesting that taxol biosynthesis is not directly regulated by miRNAs in callus cells.

Taken together, these 10 miRNAs which targeted taxol biosynthesis genes degraded 226 genes that are comprehensively involved in various primary metabolic processes and common defense activities, including “plant-hormone signaling transduction,” and “plant–pathogen interaction pathways” (Fig. 4a and b, Additional file 11).

### Biosynthesis of most secondary metabolites is upregulated but barely regulated by miRNAs

**Fig. 5:**
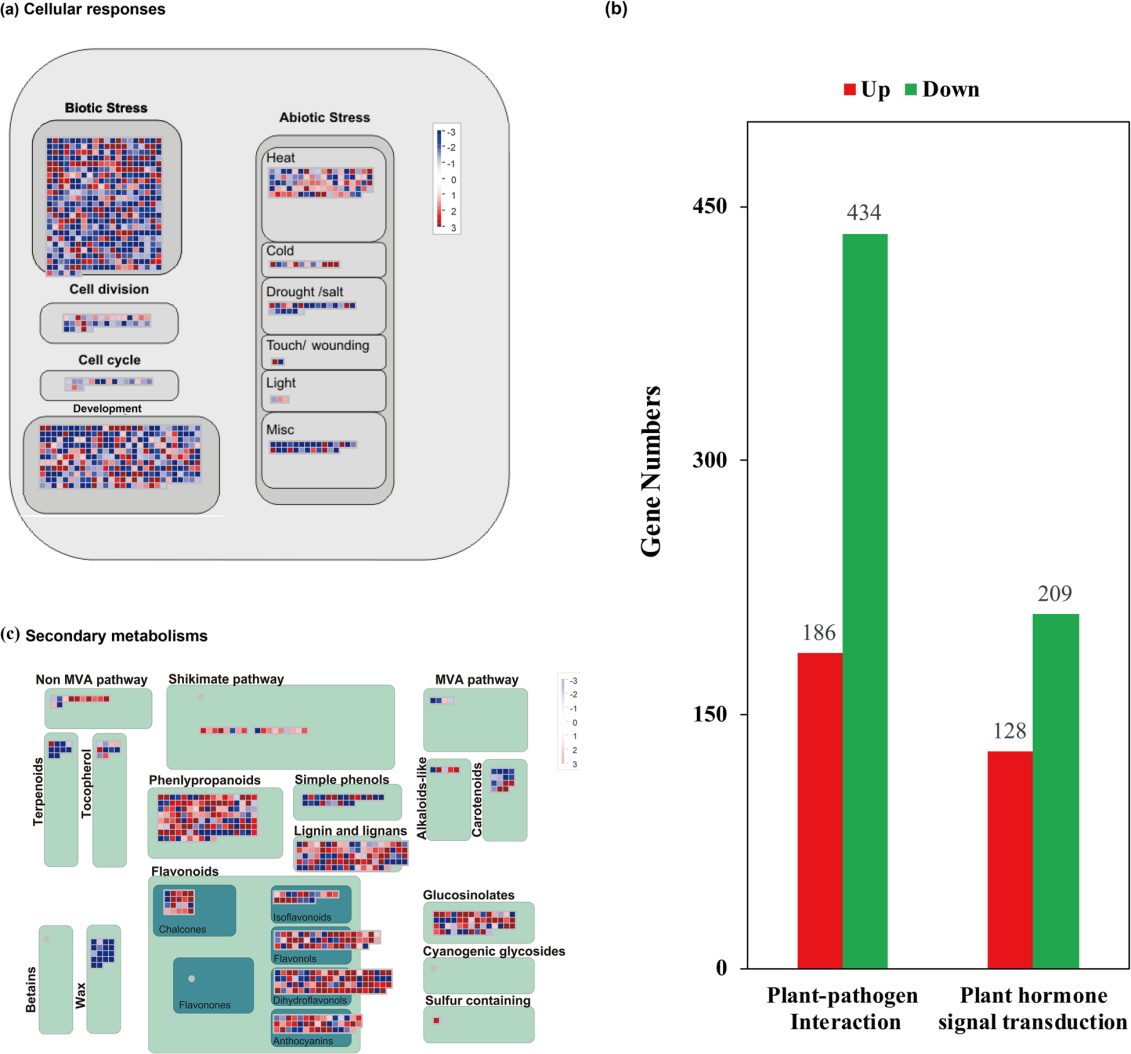
Alterations of defense activities and biosynthesis of secondary metabolites in callus cells. Callus cells altered the cellular responses (**a**), responses of Biotic- and Abiotic-stress were dominantly changed. “Plant-pathogen interaction” and “Plant hormone signaling transduction” (**b**) were crucial pathways of biotic- and abiotic-stress responses, genes of them were dominantly downregulated. Biosynthesis of secondary metabolites were mostly upregulated (**c**)

Most genes involved in the biosynthesis of secondary metabolites, especially flavonoids, phenylpropanoids, lignin, and lignans, were remarkably active in callus cells (Figs. 1c and 5d). In particular, the methylerythritol phosphate (MEP) (non- mavalonic acid (MVA)) pathway, which produces terpenoid precursors, was significantly upregulated. By contrast, the MVA pathway, which is another means to produce terpenoid precursors, was downregulated (Figs. 3a and 5d). Previous reports have indicated that MEP is a highly effective and efficient means to produce terpenoid precursors and likely a positive factor for callus cells to produce additional taxanes [23].

Only 17 DE genes of secondary metabolite biosynthesis were degraded by 15 miRNAs (Table 3). However, only five genes, namely, *PER25*, *GT4*, *CYP86A22*, *UGT85A24*, and *SNL6*, were upregulated because the four miRNAs that could degrade them were repressed (Table 3). Indeed, 598 DEGs involved in the biosynthesis of secondary metabolites were targeted by oppositely expressed miRNAs. These results indicate that miRNAs are capable of directly regulating secondary metabolism but they do not preferentially target metabolites in *T. media* callus cells, thus suggesting that a more cost-effective regulatory system for secondary metabolism should be available.

**Table 3:** Degraded DE genes involving in biosynthesis of secondary metabolites FC is short for fold change (Callus/Tissues), inf and –inf indicated they were not detected in Tissues and Callus cells respectively. AO1 (abscisic aldehyde oxidase 1), DLO2 (DMR6-like oxygenase 2), FLS (Flavonol synthase), PLR1 (Pyridoxal reductase 1), SNL6 (Cinnamoyl-CoA reductase-like SNL6), PER25 (Peroxidase 25), CBP1 (Serine carboxypeptidase 1), SDR2b (Short-chain dehydrogenase/reductase 2b) and UGT85A24 (7-deoxyloganetin glucosyltransferase)

KEGG_pathways	miRNA	log2FC (miRNA)	Gene	log2FC (Gene)
Phenylpropanoid biosynthesis	PC-5p-27911_99	−2.53	SNL6 TRINITY_DN44820_c0_g2	1.11
	smo-MIR1083-p5_2ss1TC20GC	−3.26	PER25 TRINITY_DN34416_c2_g2	6.52
	nta-MIR398-p3_2ss8AG17TC	-inf	CYP76B6 TRINITY_DN47148_c1_g1	4.62
	ata-MIR2118b-p3_2ss8CT18AT	-inf	CBP1 TRINITY_DN42316_c1_g3	1.65
Carotenoid biosynthesis	cme-MIR166e-p5_2ss9CT19GC	-inf	AO1 TRINITY_DN47212_c1_g1	1.36
Diterpenoid biosynthesis	mtr-MIR5232-p5_1ss10AG	-inf	CYP71BL1 TRINITY_DN45648_c2_g1	1.62
	mtr-MIR5232-p5_1ss9TC	-inf	CYP71BL1 TRINITY_DN45648_c2_g1	1.62
Flavonoid biosynthesis	gma-MIR4995-p5	−1.69	DLO2 TRINITY_DN34179_c0_g1	8.06
	osa-miR529b_2ss18GT20TA	-inf	FLS TRINITY_DN45365_c0_g1	1.92
	ptc-MIR2111a-p5_2ss9AT21GT	−0.84	PLR1 TRINITY_DN43111_c0_g1	3.83
Brassinosteroid biosynthesis	ath-MIR156j-p5_2ss14CT18GT	-inf	CYP720B2 TRINITY_DN47159_c3_g1	6.58
	sbi-MIR437w-p5_2ss4AG18AG	−0.62	CYP720B2 TRINITY_DN40347_c0_g2	5.03
	ath-MIR156j-p5_2ss14CT18GT	-inf	CYP720B2 TRINITY_DN42445_c0_g2	2.27
Cutin, suberine and wax biosynthesis	PC-3p-388_5924	7.12	CYP86A22 TRINITY_DN41926_c0_g1	−5.01
Monoterpenoid biosynthesis	cme-MIR166e-p5_2ss9CT19GC	-inf	SDR2b TRINITY_DN45132_c0_g1	2.81
Anthocyanin biosynthesis	smo-MIR1083-p5_2ss1TC20GC	−3.26	GT4 TRINITY_DN43852_c0_g4	7.16
Ubiquinone and other terpenoid-quinone biosynthesis	ppt-MIR1030c-p5_1ss18TG	inf	At4g27270 TRINITY_DN35770_c1_g9	−1.28
Benzoxazinoid biosynthesis	PC-3p-23175_128	−1.11	UGT85A24 TRINITY_DN45846_c2_g1	3.70

### Callus cells strengthens the metabolism of biological materials and weakens the common defense system mainly mediated by miRNAs

Among the 43 significant DE pathways found in callus cells, the pathways related to metabolism of biological substances were all strengthened (Fig. 1c, Additional file 5a, Additional file 12). For example, the metabolism of most amino acids, such as tyrosine and phenylalanine, was significantly enhanced. In addition, the DEGs of “Glycolysis” and “Citrate cycle” were remarkably upregulated; both of these pathways are key processes for decomposing sugar for living energy (Fig. 1c, Additional file 5a, Additional file 12). Interestingly, “Photosynthesis” (35 downregulated vs. 3 upregulated) and “Photosynthesis-antenna proteins” (13 downregulated) were extremely weakened (Fig. 1c, Additional file 5a, Additional file 12). This finding indicates a transformation in living behavior from autotrophic tissues to heterotrophic cells.

Degradome sequencing confirmed that degraded DEGs are significantly enriched in the metabolism of several biological materials, such as “Starch and sucrose metabolism,” “Ascorbate and aldarate metabolism,”, and “Aminoacyl-tRNA biosynthesis” (Fig. 2b). In addition, most of the degraded DEGs involved in these pathways were regulated by oppositely expressed miRNAs, thus suggesting that miRNAs effectively regulate these pathways (Fig. 2c, Additional file 13).

Biotic and abiotic stresses are the main threats to living plants; to address these stresses, plants execute a number of response activities, such as “Plant–pathogen interaction,” “Plant hormone signal transduction,” “Phagosome,” and “Endocytosis” [24–30]. Callus cells showed more downregulated genes involved in abiotic and biotic stress responses compared with tissues but showed upregulated heat/cold/light responses (Fig. 5a). For “Plant–pathogen interaction” and “Plant hormone signal transduction”, 70% (434) and 62% (209) DEGs were downregulated (Fig. 5b and c, Additional file 5a). For example, *jasmonic acid-amino synthetase 1* (*JAR1*) and *nonexpresser of pathogenesis-related 1* (*NPR1*), which positively function in JA- and SA-signaling transduction, were significantly downregulated (Additional files 14 and 15). These results suggest that callus cells are fragile in terms of stress response and disease resistance.

MiRNAs are the main factors regulating common defense activities and significantly enriched in “Plant–pathogen interaction” “Plant hormone signal transduction”, and “Endocytosis” [31]. Among the pathways observed, “Plant–pathogen interaction” and “Plant hormone signal transduction” were the most enriched pathways regulated by miRNAs; indeed, 77 and 59 DE degraded targets of pathogen interaction and plant hormone signal transduction were respectively detected in callus cells (Fig. 2b, Additional file 10). In addition, degraded DE targets were detected in “Phagosome”, “Spliceosome” and “Base excision repair”, which are other pathways related to defense. Such findings indicate that miRNA is an important regulator of these primary metabolism pathways and common defense activities. In particular, pathways, such as “plant–pathogen interaction” and “plant hormone signal transduction”, are the prior regulation targets of miRNAs.

### TFs are important targets of miRNAs

**Fig. 6:**
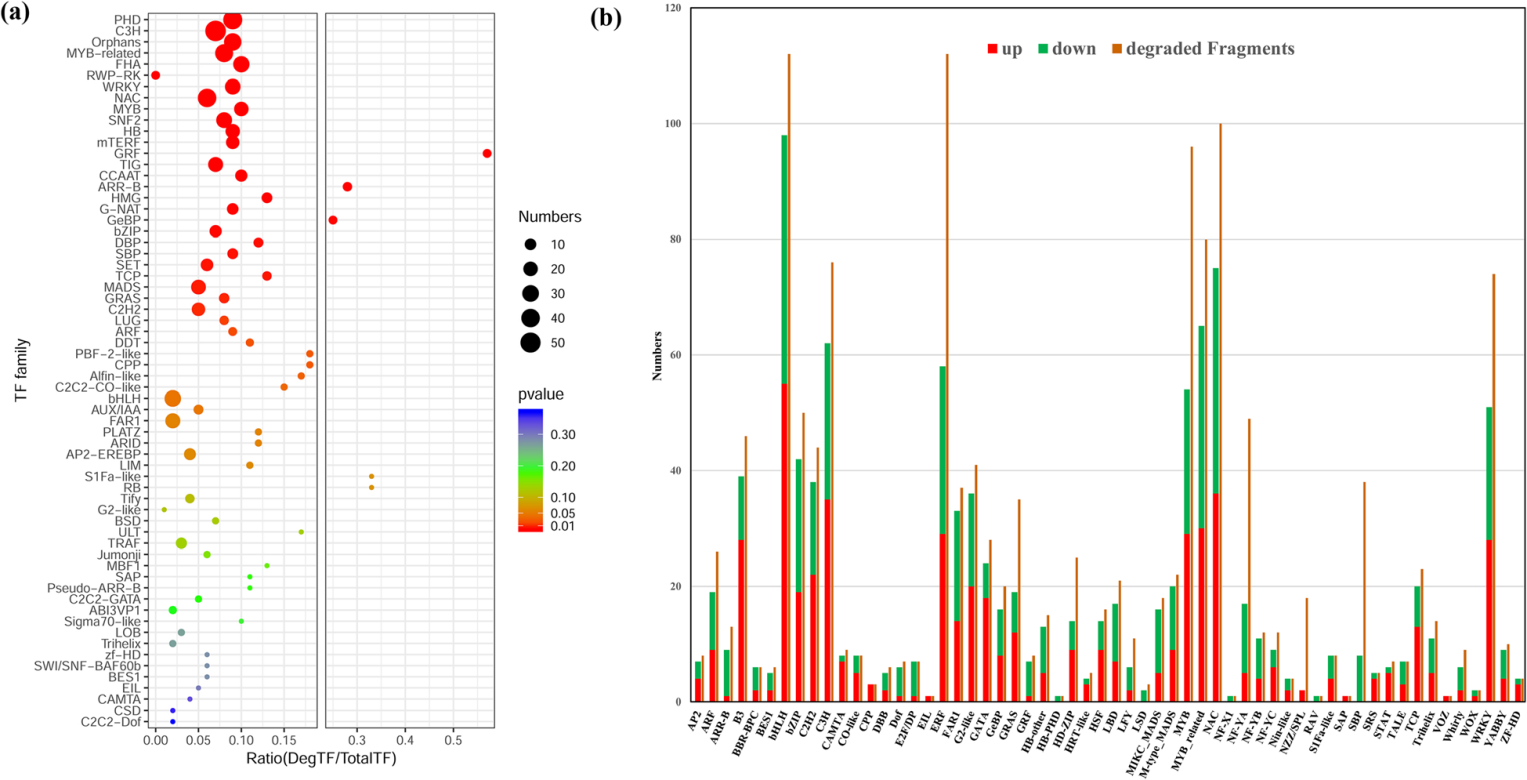
Transcription factors were important targets of miRNAs. More than half of TF families were significantly degraded by miRNAs (**a**), and several TFs were degraded by more than one miRNAs so that degraded fragments were much more than the DE TFs, suggesting these TFs played crucial roles in transcriptional reprogramming in callus cells (**b**)

Among 1830 degraded targets, 635 (34.7%) were TFs and various transcriptional regulators and degraded by 236 miRNAs, thus constituting 894 pairs of miRNA–TF modules (Additional file 9). Among the 635 degraded TFs, PHD, C3H, bHLH, WRKY, MYB, and NAC were mostly regulated by miRNAs (Fig. 6a). A total of 156 miRNAs degraded more than one TF, and cme-MIR166e-p5_2ss9CT19GC degraded 71 TFs (Fig. 6b, Additional file 16). A total of 426 miRNA-TF modules showed contrasting expression patterns, and 292 pairs (68.6%) were constituted by downregulated miRNA and upregulated TF, thus indicating that callus cells repress the expression of miRNAs to regulate bioactivities (Additional file 16). Previous reports also concluded that miRNAs repress the expression of most genes until these genes are needed [32].

Among the TFs, ERF, bHLH, NAC, and MYB had the highest number of degraded fragments because one TF were degraded by several miRNAs, leading to a higher number of degraded fragments than DEGs (Fig. 6b). ERF, SBP, NZZ/SPL, and NF-YA were the most enriched targets of degradation, thus suggesting their importance in transcriptional reprograming from tissues to callus cells (Fig. 6b).

**Fig. 7:**
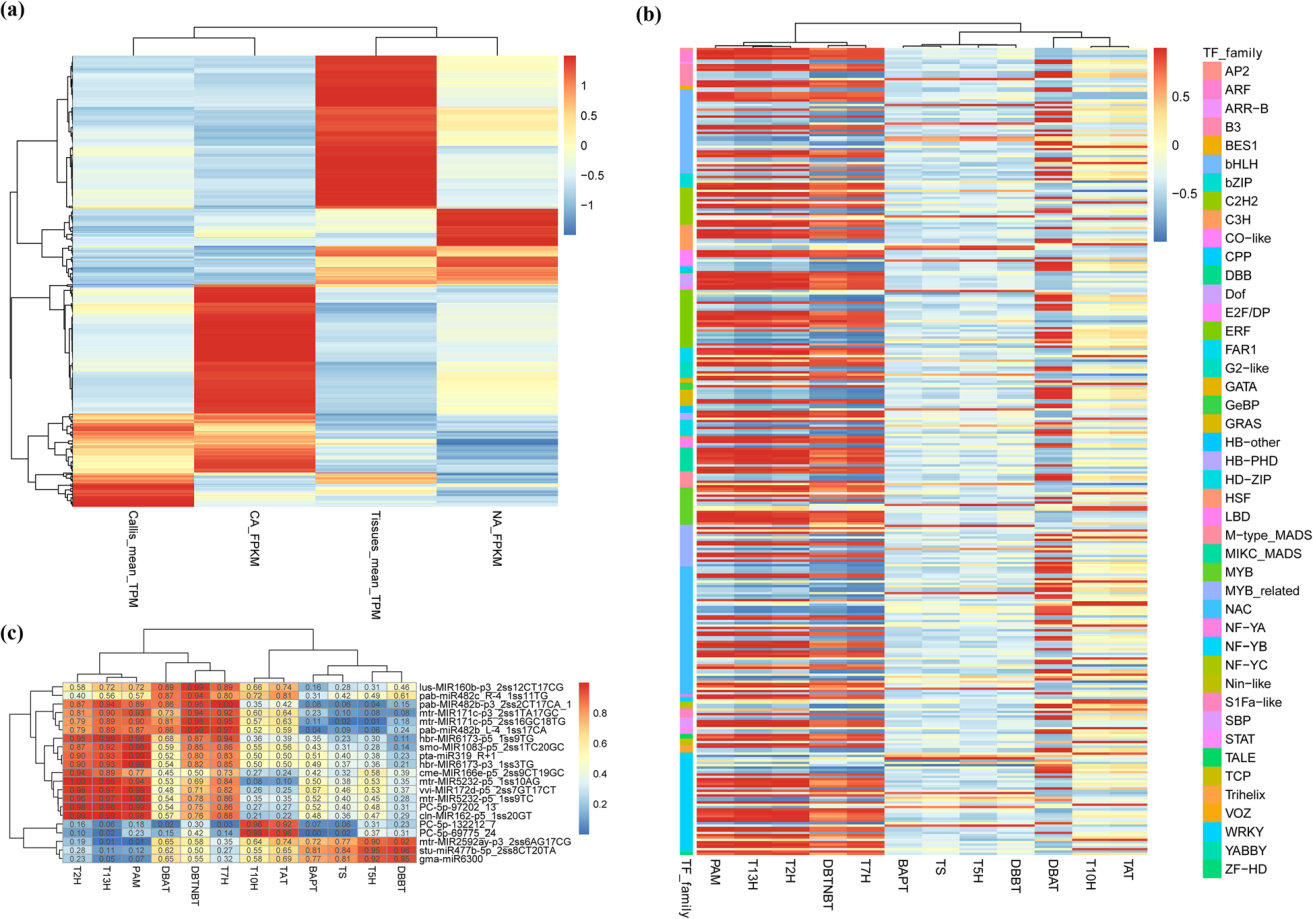
Candidate TFs and miRNAs related to regulation of taxol biosynthesis. Previously, transcriptional profiles during long-term subculture process were detected. Thus, integrative analysis of these expression profiles with Callus&Tissues were helpful to identify the key transcription factors. All DE TFs were analyzed their expression patterns comparing with 12 known taxol biosynthesis genes (**a**), and the co-expression relation values of candidate TFs were showed in (**b**). The candidate miRNAs and co-expression values were showed in (**c**)

The roles of these TFs were analyzed here on the basis of studies on several known *Taxus* TFs [33–36]. TcERF15, TcMYC2a, TcJAMYC1/2/4, and TcWRKY1/8/20/26/47/52 were highly correlated with taxol biosynthesis genes with Pearson coefficients greater than 0.9 (Fig. 7b, Additional file 17). Interestingly, 4 homologues, TcMYC2a and TcJAMYC1/2/4, showed contrasting expression patterns in callus cells. These results are highly consistent with results from functional studies. TcMYC2a from *Taxus chinensis* and TcJAMYC1/2/4 from *Taxus cuspidata* function as positive and negative regulators in taxol biosynthesis, respectively [34].

### Candidate TFs and miRNAs involved in the regulation of taxol biosynthesis

To determine candidate TF regulators, Pearson coexpression correlations were analyzed in combination with the previous dataset of long-term subcultured *T. chinensis* cells [7]. A total of 451 TFs were similarly or oppositely expressed with taxol biosynthesis genes (Fig. 7a), and 346 of 451 TFs were highly correlated with taxol biosynthesis genes with coefficient values greater than 0.9 (Fig. 7b, Additional file 17). *T13H*, *T2H*, and *PAM* were correlated with 161, 145, and 141 TFs, respectively. Although *DBAT* and *T7H* were correlated with 107 TFs, they were simultaneously correlated with only 38 TFs (Fig. 7b, Additional file 17). TF families, NAC, WRKY, bHLH, and ERF were the most coexpressed candidate regulators.

Among the 346 miRNAs mediating TF degradation, the expression patterns of only 28 miRNAs were similar to those of taxol biosynthesis genes. Subsequent coexpression analysis confirmed that 21 miRNAs were closely related to taxol biosynthesis with high coefficient values (> 0.9 or < − 0.9). Nine taxol biosynthesis genes, namely, *T5H*, *TAT*, *T10H*, *DBBT*, *DBTNBT*, *T7H*, *T13H*, *PAM*, and *T2H*, were related to miRNAs. *PAM* and *T13H* were mainly related to 10 miRNAs; this relationship indicates that *PAM* and *T13H* are crucial regulatory targets. Hbr-MIR6173-p5_1ss9TG was coexpressed with 4 taxol biosynthesis genes. In addition, a novel miRNA first identified in *Taxus*, PC-5p-97202_13, simultaneously targeted *DBBT* and *DBTNBT;* this characteristic suggests its important regulatory roles in taxol biosynthesis (Figs. 4c, d, and 7c). Although these coexpressed miRNAs do not degrade taxol biosynthesis genes, they may regulate taxol biosynthesis through other means.

## Discussion

Besides taxol, the biosynthesis of most secondary metabolites, such as flavonoids and phenylpropanoids, but not that of waxes, is remarkably enhanced in callus cells (Figs. 1c, 5d). Secondary metabolites, flavonoids, phenylpropanoids, and taxol are often used as defense materials in plants [37–39]. However, common defense activities, such as “Plant–pathogen interaction” and “Plant hormone signaling transduction”, are significantly downregulated (Fig. 5, Additional file 5).

“Plant–pathogen interaction” and the JA/SA-signaling pathways help plants defend themselves against pathogens and external stimuli [26, 40]. Our results showed that callus cells dramatically downregulate the genes of these pathways, such as JAR1 and NPR1, which are positive regulators of the JA- and SA-signaling pathways, respectively (Additional files 5 and 15). Moreover, although several pathways did not show a significant change in callus cells, they tended to be downregulated. A total of 150 (81.7%) DEGs of “Endocytosis,” 32 (82.1%) DEGs of “Phagosome,” and 23 (71.8%) DEGs of “Regulation of autophagy” were remarkably downregulated in callus cells (Additional file 5). Downregulation of these pathways indicates that callus cells become fragile in response to external stimuli. The pathways, “Nucleotide excision repair” (18 genes of 23 DEGs were downregulated), “Base excision repair” (15 down, 5 up), “Ubiquitin mediated proteolysis” (25 down, 12 up), and “Spliceosome” (76 down, 34 up) were also downregulated; this phenomenon suggests that callus cells may be susceptible to genetic mutagenesis in vivo (Additional file 5). In addition, transduction of auxin- and cytokinin-signals were inhibited because the corresponding receptors and transducing factors were downregulated, possibly explaining the slow growth rate of callus cells (Additional files 14 and 15).

According to these results of increasing of biosynthesis of secondary metabolites and reducing of defense pathways, callus cells appeared to use secondary metabolites for defense instead of common defense pathways. Given that callus cells transition to heterotrophic feeding on substances in the medium, this alteration may be related to cost-effectiveness. Specifically, “Photosynthesis” was completely downregulated in callus cells, and the metabolism of several sugars and that of almost all amino acids contained in medium were enhanced. Rojas et al. indicated that the downregulation of some primary metabolic processes, such as photosynthesis, could save energy and promote defense responses; they also revealed that the upregulation of amino acid metabolism contributes to plant defenses [37]. Moreover, secondary metabolites could be used for immediate defense materials, whereas common defense pathways, such as “Plant-pathogen Interactions” and “JA/SA signaling transduction”, require time to respond. Thus, accumulation of secondary metabolites is a cost-effective strategy for survival.

This inference is supported by a previous study on *T. chinensis* cells subjected to 10 years of subculture. In long-term subcultured cells, the metabolism of biological substances is enhanced, whereas common defense activities are attenuated. Remarkable reductions in taxol and other secondary metabolites suggest that long-term subculture cells abandon unnecessary defense activities [7]. These findings indicate that taxol accumulation is a survival strategy that incurs extra costs for wild plants. Thus, callus cells would prefer to choose a defense system with increased cost effectiveness.

MiRNAs are the tools used by callus cells to reprogram their metabolism. RNAs play decisive roles in changes in primary metabolism and defense systems, including biological substance metabolism, “plant–pathogen interactions”, and “Endocytosis.” Although miRNAs did not regulate the secondary metabolism of callus cells, they degraded a large number of TFs, such as MYB, ERF, bHLH, and other factors related to the biosynthesis of secondary metabolites. Moreover, a growing number of studies have revealed that regulation by miRNA-TFs is a main function mode in plants [11, 41, 42].

## Conclusion

**Fig. 8:**
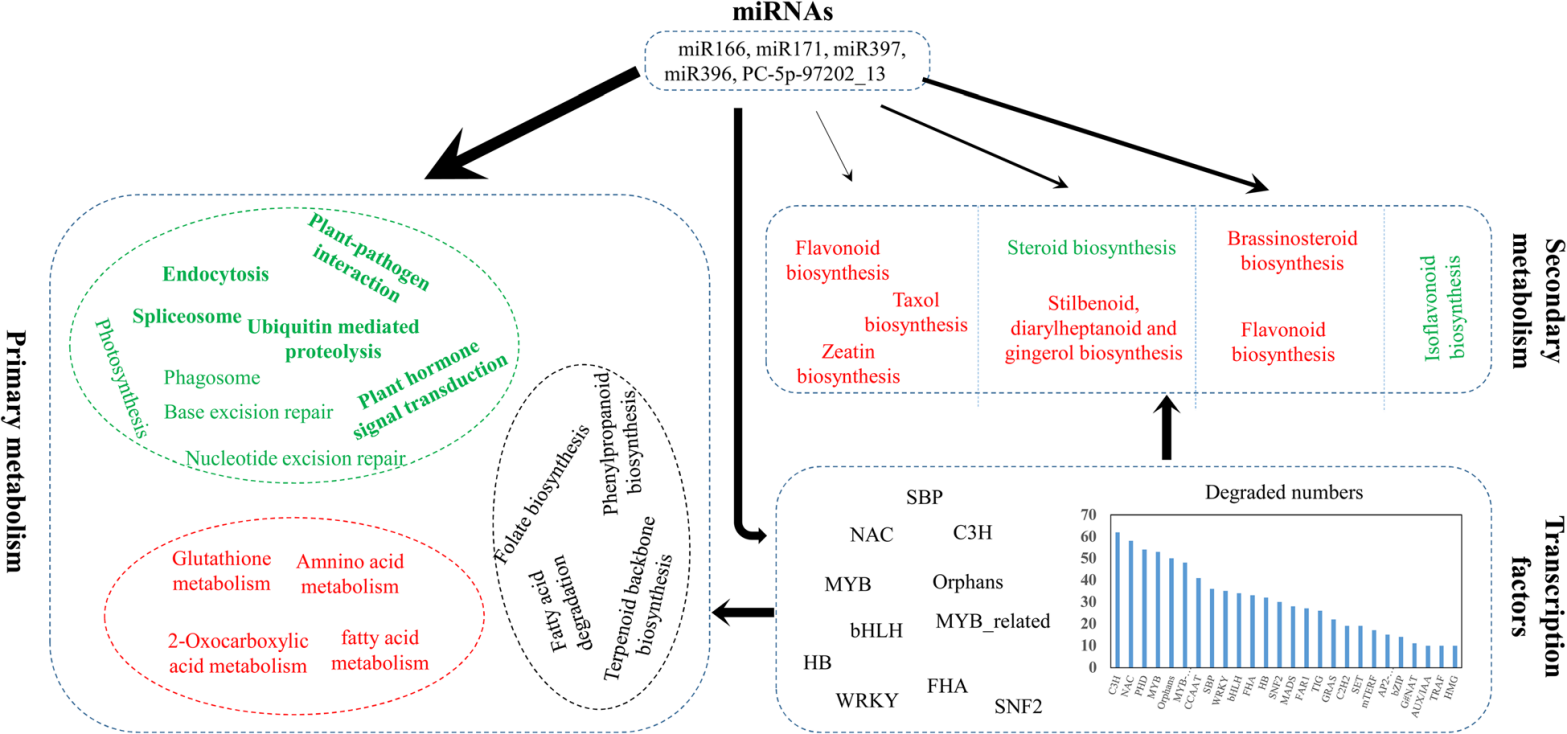
miRNA regulation models during Callus formation and long-term subculture. The regulation model of miRNA on transcriptional reprogramming in Callus cells. All the pathways in Figure were significantly differentially expressed, and the thickness of the arrows indicated the regulation strength by miRNAs. Some pathways also significantly changed but they were not regulated by miRNA, such as isoflavonoid biosynthesis. The pathways in green indicated most DEGs of these pathways were downregulated, red indicated most DEGs were upregulated, black means the upregulated genes of these pathways were as much as the downregulated ones. Most pathways of secondary metabolism were barely regulated by miRNAs directly, probably the regulation model was a “miRNA-TF-enzyme genes” mode

Newly induced *Taxus* callus cells exhibited reprogramed transcriptional profiles to survive and adopt to novel situations (Fig. 8). Increasing accumulation of taxol and other secondary metabolites appeared to be an active mode to compensate for weakened immune and defense systems because callus cells repress pathways such as “Plant–pathogen interaction” and “JA/SA–signaling transduction”, which are crucial for plants to defend themselves against stresses. In addition, callus cells repressed activities such as “nucleotide/protein mismatch repair” and “spliceosome” but reinforced the metabolism and biosynthesis of biological substances. These alterations suggest that callus cells prefer a cost-effective survival strategy. Subsequent analysis revealed that miRNAs are key tools for the transcriptional reprogramming of callus cells. The reduction of defense and immune systems and increased metabolism and biosynthesis of essential substances mainly depended on miRNAs. Although a small proportion of the genes involved in the biosynthesis of secondary metabolites were degraded by miRNAs, miRNAs regulated secondary metabolism by degrading a series of TFs. Several candidate TFs were highly correlated and coexpressed with taxol biosynthesis genes, and some TFs were regulated by miRNAs. Our results reveal the candidate regulators of TFs and miRNAs for taxol biosynthesis, as well as the significance of taxol biosynthesis for plant regulation networks.

## Methods

### Plant materials and callus induction

The 4-years-old *Taxus media* Rehder, obtained from Hubei green tide biotechnology company, were cultivated from cuttings at nursery in Huazhong University of Science and Technology. The tissues were fresh stems and callus cells were induced with MS medium containing 0.3 mg/L 2,4-D, 0.4 mg/L NAA from the fresh stems from the same plant, these callus cells were maintained for 6 months with 62# (modified Gamborg’s B5, the hormones concentration were 0.5 mg/L NAA, 0.5 mg/L 6-BA and 0.2 mg/L 2.4-D) medium at 25 °C without daylight [43]. Six months culturing made callus cells form a stability growth state so that it was beneficial for us to screen the key regulate factors.

### High-throughput mRNA sequencing, assembly and differential expression genes screening

Total 6 samples belong to two groups (named as Tissues and Callus) were used to detect their transcriptional profiles. Total RNA isolation and library construction were conducted by LC-bio Tech (Hangzhou, China). The average insert size for the paired-end libraries was 300 bp (±50 bp), then the paired-end sequencing was performed on an Illumina Hiseq 4000 (1 × 150 bp for each end) platform following the vendor’s recommended protocol. Firstly, Cutadapter V1.9 and fqtrim V0.94 were used to remove the reads that contained adaptor contamination, low quality bases and undetermined bases [44]. Then FastQC V0.10.1 was used to verify sequence quality (http://www.bioinformatics.babraham.ac.uk/projects/fastqc/) including the Q20, Q30 and GC-content of the clean data. All downstream analyses were based on clean data of high quality, which did not contain any adaptor contamination (if the read was smaller than 100 bp after removing the adapter, the read were deleted), low quality bases (if the average quality of every 6 bp of single read is lower than 20, the entire 3′-flanking sequence was deleted) and undetermined bases (if the unknown base is more than 5% in a read, the read will be deleted). De novo assembly of the transcriptome was performed with Trinity 2.4.0. All assembled Unigenes were aligned against the non-redundant (Nr) protein database (http://www.ncbi.nlm.nih.gov/), Gene ontology (GO) (http://www.geneontology.org), SwissProt (http://www.expasy.ch/sprot/), Kyoto Encyclopedia of Genes and Genomes (KEGG) (http://www.genome.jp/kegg/) and eggNOG (http://eggnogdb.embl.de/) databases using DIAMOND [45] with a threshold of E-value<1e-5. Salmon was used to perform expression level for Unigenes by calculating TPM [46], and R package edgeR was applied for calculating statistical significance (*p*-value), then FDR were used to detected the false rate [47]. Since there were three independent bio-repeats of each individual group, the differentially expressed unigenes were selected not only with |log2 (fold change)| > 1, but also with FDR < 0.05. The GO and KEGG enrichment analysis of DEGs were validated by Fisher’s Exact Test with a cutoff *p*-value< 0.05.

### MiRNA analysis and targets prediction

Part of total RNAs from the same 6 samples of transcriptome were also used to conduct library for miRNA-seq. The miRNA library was constructed with TruSeq Small RNA Sample Prep Kits (Illumina, San Diego, USA), and they were single-end sequenced by Illumina Hiseq2000/2500 with 1 × 50 bp. ACGT101-miR (LC Sciences, Houston, Texas, USA) was used to process raw reads by removing adapter dimers, junk (unknown nucleotide bases more than 10%), low complexity, common RNA families (rRNA, tRNA, snRNA, snoRNA) and repeats as described by Hu et al., 2018 [48]. Subsequently, unique sequences with length in 18~25 nucleotide were mapped to specific species precursors in miRBase 21.0 by BLAST search to identify known miRNAs that allowed length variation at both 3′ and 5′ ends and one mismatch inside of the sequence. For novel miRNAs, the unmapped sequences were BLASTed against the shallow sequencing *Taxus baccata* genomes [49], and the hairpin RNA structures containing sequences were predicted from the flank 120 nt sequences using RNAfold software (http://rna.tbi.univie.ac. at/cgi-bin/RNAfold.cgi).

Firstly, expression levels of miRNAs were based on normalized deep-sequencing counts, the normalization procedures were according to Zhao et al., 2017 [50]. Differential expression of miRNAs was analyzed by using Student T-test, when the p-value was less than 0.05, the miRNAs were considered as differentially expressed miRNAs [50] (Additional file 18).

To predict the genes targeted by most abundant miRNAs, Target Finder ver. 50 were used to identify miRNA binding sites using default settings [51].

### Degradome sequencing and data processing

Approximately 20 μg total RNA that mixed from 6 samples were used to prepare degradome library followed as with some modification [52]. Then, the libraries were sequenced using the 5’adapter only, resulting in the sequencing of the first 36 nucleotides of the inserts that represented the 5′ ends of the original RNAs. And the single-end sequencing (36 bp) were performed on an Illumina Hiseq2500.

After removing adaptors and low quality reads, the extracted sequencing reads were then used to identify potentially cleaved targets by the CleaveLand pipeline [53]. The degradome reads were mapped to the transcripts obtained above. Only the perfect matching alignment(s) for the given read would be kept for degradation analysis. All resulting reads (t-signature) were reverse complemented and aligned to the miRNA identified in our study. All the identified targets were subjected to BlastX analysis to search for similarity, and then to GO analysis to uncover the miRNA-gene regulatory network on the basis of biological process, cellular module and molecular function.

### Taxanes extraction and quantitative analysis

The tissues and callus cells were firstly grinding with liquid Nitrogen, then taxanes were extracted using the method described previously [54]. And the quantitative analysis was conducted using HPLC as the previous reports [36].

### Transcription factors and transcriptional regulators identification

Transcription factors and transcriptional regulators annotation were conducted by blasting the assembling unigenes against plnTFdb V3.0 (http://plntfdb.bio.uni-potsdam.de/v3.0/) with the E-values <1e-5. Expression patterns were drawn by Morpheus (https://software.broadinstitute.org/morpheus/) with defaulting parameters, and Pearson co-expression coefficient values were analyzed by CORREL formula within Excel 2016 [55, 56].

## Supplementary information

**Additional file 1. **Information of RNA-seq results The length distribution of assembled unigenes was showed in (a). Species distribution by annotated with NR was showed in (b), *Picea sitchensis* had the most homologues of *Taxus media*. (c) eggNOG annotation of all unigenes. (d) Expression volcano map of unigenes. (e) Expression patterns of all DEGs.

**Additional file 2. **Information of miRNA-seq The length distribution of miRNAs was showed in (a). Total and first nucleotide bias were showed in (b) and (c) respectively. (d) Pearson relation analysis of all expressed miRNAs between six samples. (e) Venn diagram of miRNAs in callus cells and tissues. (f) Expression patterns of all DE miRNAs (*p* < 0.05).

**Additional file 3.** Annotation and expression levels of assembled unigenes.

**Additional file 4.** All expressed miRNAs All expressed miRNAs were listed in sheet (a) including known and novel miRNAs. The sheet (footnotes) provided the information about the table.

**Additional file 5.** GO annotations and statistics of DEGs GO statistic of DEGs (a) and GO annotation of DEGs (b). The genes with only one specific annotation was counted.

**Additional file 6.** GO annotation of DEGs and degraded DEGs Supplement figure compared the GO annotations of DEGs (a and b) with degraded DEGs (c and d). (a) GO annotations of DEGs, (b) GO enrichment analysis of DEGs (first 20 terms). (c) GO annotations of degraded DEGs, (d) GO enrichment analysis of degraded DEGs (first 20 terms). All these results suggested that miRNAs comprehensively regulated in transcriptional reprogramming in callus cells.

**Additional file 7.** Alteration maps of several important bioactivities Several important bioactivities were listed here: (a) biotic- and abiotic-stress responses, (b) regulators, (c) large enzyme families, (d) Plant-hormone transduction and (e) overview of cell functions. These drawing were made by Mapman, and the boxes indicated genes, red means upregulated and blue was down regulated.

**Additional file 8.** KEGG annotations and statistics of DEGs KEGG statistic of DEGs (a) and KEGG annotation of DEGs (b). The genes with only one specific annotation was counted.

**Additional file 9.** DE miRNAs and their expression levels.

**Additional file 10.** Degradome-seq results All the degraded targets and miRNAs were listed. Y in column (DegradomeDetection) means the degraded fragment was sequenced, while N indicated the target was only predicted by software TargetFinder.

**Additional file 11.** Statistics of KEGG annotations The KEGG annotations of degraded DEGs were validated along with the DEGs, and the results showed that among 52 DE KEGG pathways, 19 were significantly regulated by miRNAs.

**Additional file 12 ***Taxus media* mapman database annotations.

**Additional file 13.** Ten miRNA regulatory networks which targeted to taxol biosynthesis genes.

**Additional file 14.** Targets of 10 miRNAs which targeted to taxol biosynthesis genes All degraded targets of 10 miRNAs were listed.

**Additional file 15.** DEGs related to Plant Hormone Signaling Transduction All DEGs that related to “Plant Hormone Signaling Transduction” were listed. BR was short for Brassinosteroid, SA short for Salicylic acid, GA short for Gibberellin acid, ABA short for Abscisic acid, JA short for Jasmonic acid and ET short for Ethylene.

**Additional file 16.** Degraded transcription factors All degraded transcription factors and related miRNAs were listed in (a). Transcription factors were annotated with plnTFdb database.

**Additional file 17.** Co-expression values of TFs with taxol biosynthesis genes The co-expression correlation co-efficiency values were displayed.

**Additional file 18:.** miRNA norization method.

## Data Availability

All the raw reads of mRNA-, miRNA-seq and degradome data were submitted to SRA (Sequence Read Archive, NCBI, https://www.ncbi.nlm.nih.gov/sra/) under the Bioproject number PRJNA497757, the accession numbers are SRR8083193 to SRR8083198.

## Abbreviations

AO1: Abscisic aldehyde oxidase
BAPT: Phenylpropanoyltransferase
BIII: Baccatin III
DAB III: 10-Deacetyl Baccatin III
DBAT: 10-deacetylbaccatin III-10-O-acetyl transferaseferase
DBBT: Taxane 2-alpha-O-benzoyltransferase
DBTNBT: 3′-N-debenzoyltaxol N-benzoyltransferase
DLO2: DMR6-like oxygenase 2
FLS: Flavonol synthase
JA: Jasmonic acid
JAR1: Jasmonic acid-amino synthetase 1
NPR1: Nonexpresser of pathogenesis-related 1
PAM: Phenylalanine ammonia-lyase
PLR1: Pyridoxal reductase 1
SA: Salicylic acid
T10H: 5-alpha-taxadienol-10-beta-hydroxylase
T13H: 13-alpha-hydroxylase gene
T2H: Taxane 2-alpha hydroxylase
T5H: Taxadiene 5-alpha hydroxylase
TASY: Taxadiene synthase
TAT: Taxadienol acetyl transferase
TF: Transcription factors
