# Supplementary figures and images for: Transcriptional reprogramming strategies and miRNA-mediated regulation networks of Taxus media induced into callus cells from tissues
Source: BMC Genomics. 2020 Feb 18;21:168. doi: 10.1186/s12864-020-6576-2 (PMC7029464; doi:10.1186/s12864-020-6576-2)

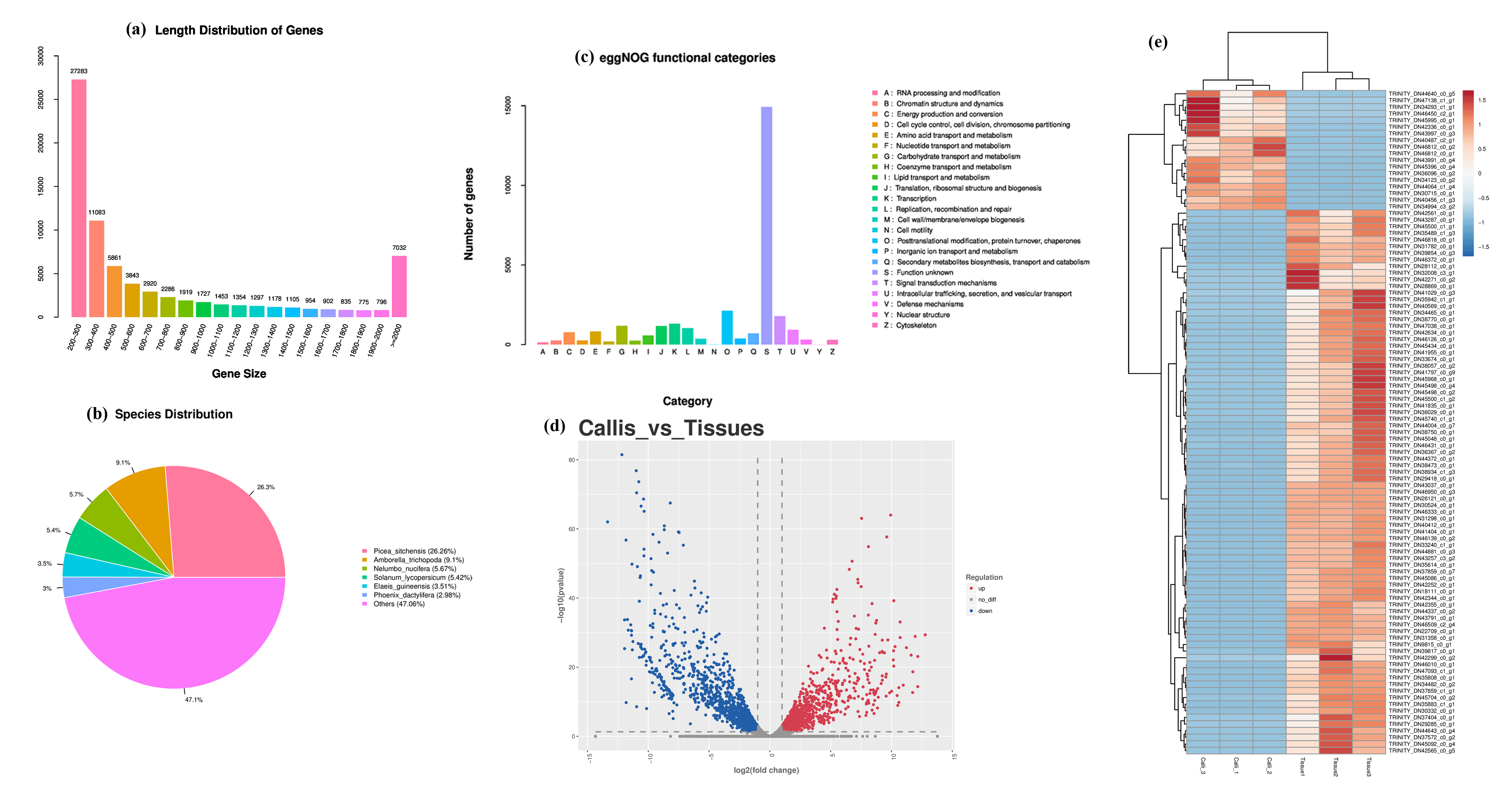

Supplement: Supplementary file 1 — Additional file 1. Information of RNA-seq results The length distribution of assembled unigenes was showed in (a). Species distribution by annotated with NR was showed in (b), Picea sitchensis had the most homologues of Taxus media. (c) eggNOG annotation of all unigenes. (d) Expression volcano map of unigenes. (e) Expression patterns of all DEGs. [file 12864_2020_6576_MOESM1_ESM.tif]

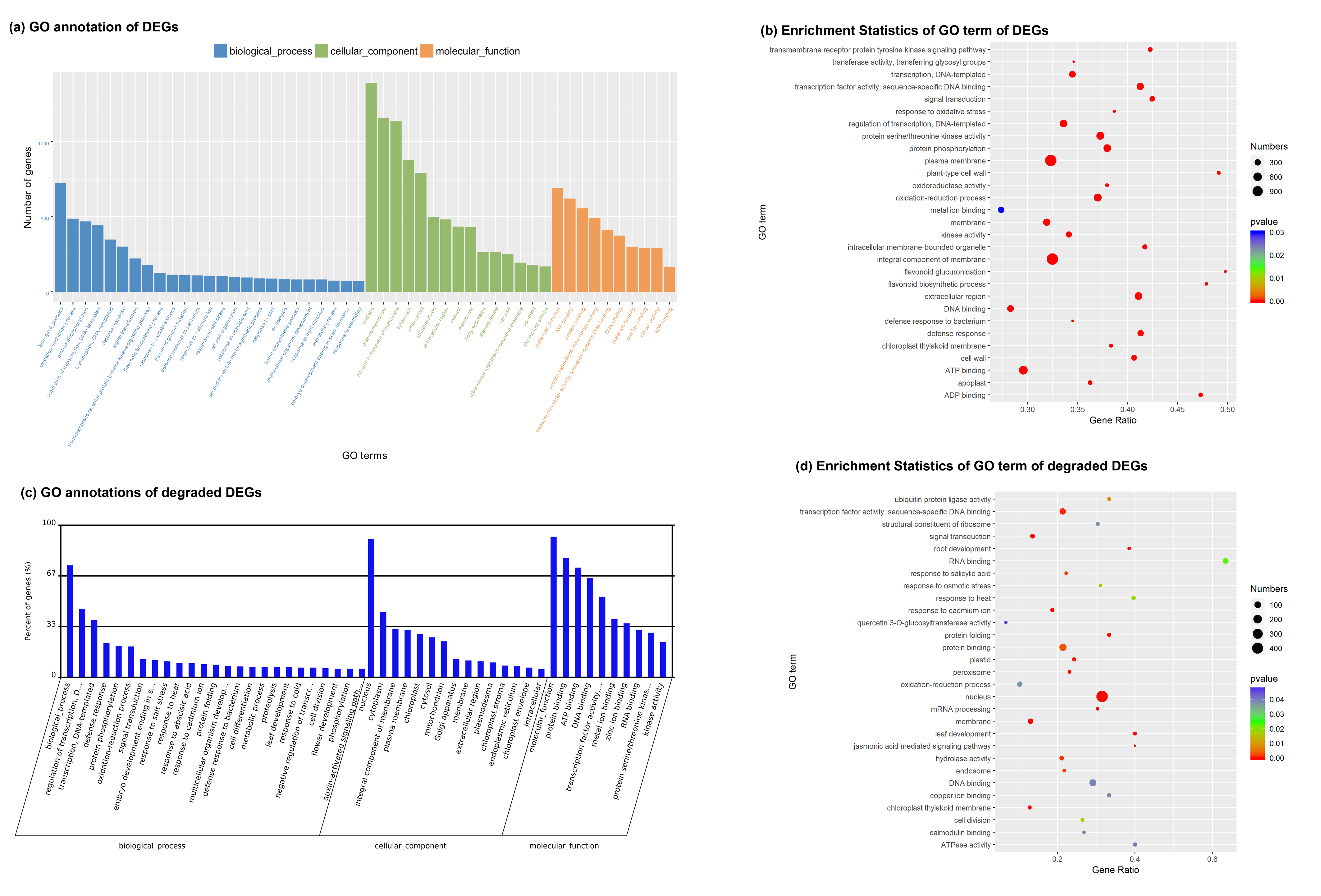

Supplement: Supplementary file 3 — Additional file 3. Annotation and expression levels of assembled unigenes. [file 12864_2020_6576_MOESM3_ESM.tif]

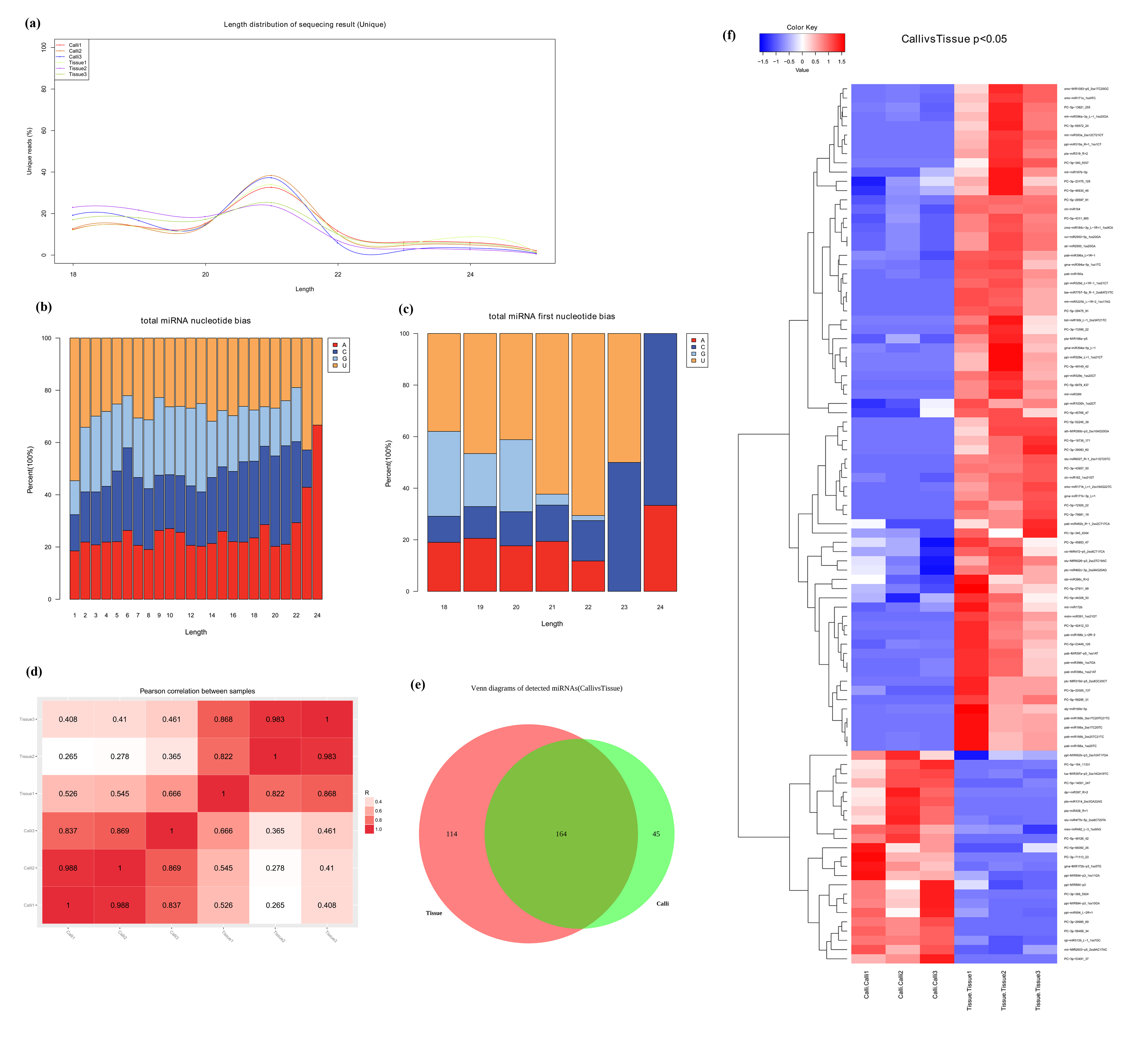

Supplement: Supplementary file 7 — Additional file 7. Alteration maps of several important bioactivities Several important bioactivities were listed here: (a) biotic- and abiotic-stress responses, (b) regulators, (c) large enzyme families, (d) Plant-hormone transduction and (e) overview of cell functions. These drawing were made by Mapman, and the boxes indicated genes, red means upregulated and blue was down regulated. [file 12864_2020_6576_MOESM7_ESM.tif]

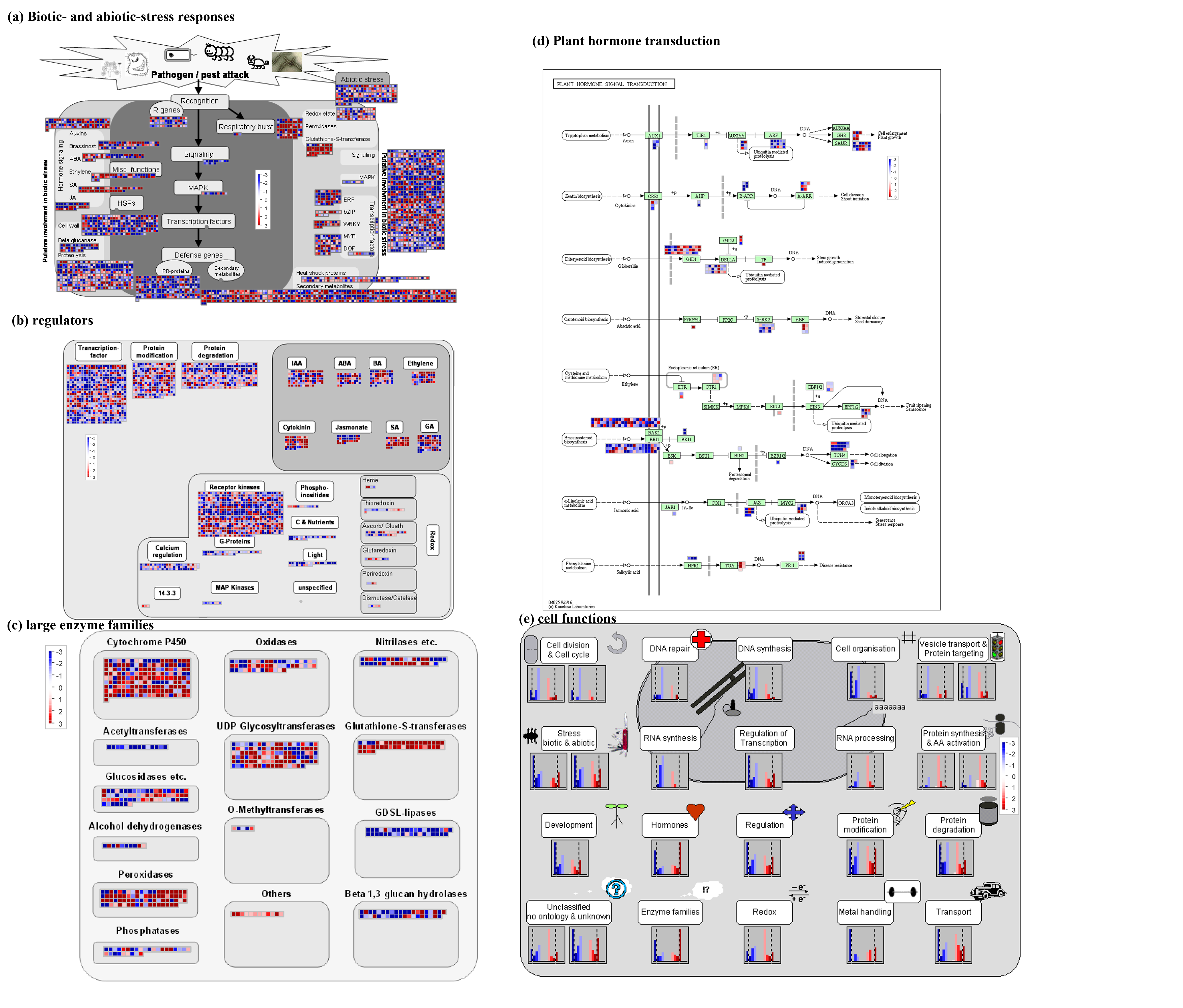

Supplement: Supplementary file 14 — Additional file 14. Targets of 10 miRNAs which targeted to taxol biosynthesis genes All degraded targets of 10 miRNAs were listed. [file 12864_2020_6576_MOESM14_ESM.tif]
