# Supplementary material for: Transcriptional reprogramming strategies and miRNA-mediated regulation networks of Taxus media induced into callus cells from tissues
Source: BMC Genomics. 2020 Feb 18;21:168. doi: 10.1186/s12864-020-6576-2 (PMC7029464; doi:10.1186/s12864-020-6576-2)
Supplement: Supplementary file 18 — Additional file 18:. miRNA norization method. [file 12864_2020_6576_MOESM18_ESM.docx]

**COPY NUMBER NORMALIZATION IN SMALL RNA SEQUENCING DATA ANALYSIS**

A modified global normalization is used to correct copy numbers among different samples. Basic assumptions and procedures involved in this method are described below.

**Assumptions**

1. There is a subset of sequences of a significant number that do not change significantly across all samples. The following normalization method is a modified global normalization method which is valid under this assumption.

2. In sequencing measurements, experimental condition variations may lead to copy-number reading variations. However, in each sample run the reading variations occur in the same proportion to all sequences. This assumption permits the use of a single correction factor for all sequences in a sample.

**Procedure**

1. Find a common set of sequences among all samples

2. Construct a reference data set. Each data in the reference set is the copy number median value of a corresponding common sequence of all samples.

3. Perform 2-based logarithm transformation on copy numbers ( log2 ( copy#) ) of all samples and reference data set.

4. Calculate the log2 (copy#) difference ( Δ log2 ( copy#) ) between individual sample and the reference data set.

5. Form a subset of sequences by selecting |Δ log2 ( copy#) |<2, which means less than 4 fold change from the reference set.

6. Perform linear regressions between individual samples and the reference set on the subset sequences to derive linear equations y=a_i_x+b_i_ where a_i_ and b_i_ are the slop and interception, respectively, of the derived line, x is log 2 (copy#) of the reference set, and y is the expected log 2 ( copy# ) of sample i on a corresponding sequence.

7. Calculate the mid value $x_{\mathrm{mid}}\frac{\max x\min x}{2}$ of the reference set. Calculate the corresponding expected log 2 (copy#) of sample i y_i,mid_= aix_mid_+bi. Let y_r,mid_ = x_mid_. Let Δyi=y_r,mid_ − y_i,mid_, which is the logarithmic correction factor of sample i. We then derive the arithmetic correction factor fi =2^Δyi^ of sample i.

8. Correct copy numbers of individual samples by multiplying corresponding arithmetic correction factor f_i_ to original copy numbers.
